# Correction: de Forges de Parny et al. Towards a Global Scale Quantum Information Network: A Study Applied to Satellite-Enabled Distributed Quantum Computing. *Entropy* 2025, *27*, 1166

**DOI:** 10.3390/e28101072

**Published:** 2026-09-30

**Authors:** Laurent de Forges de Parny, Luca Paccard, Mathieu Bertrand, Luca Lazzarini, Valentin Leloup, Raphael Aymeric, Agathe Blaise, Stéphanie Molin, Pierre Besancenot, Cyrille Laborde, Mathias van den Bossche

**Affiliations:** 1 Thales Alenia Space, 26, Avenue J-F Champollion, 31037 Toulouse, France; 2 Thales SIX GTS, 4 Avenue des Louvresses, 92230 Gennevilliers, France

**Text Correction**

There was an error in the original publication [1]. This was just a minor typo when writing the equation $\eta^{Alice}=\eta^{Bob}{=R}_{src}{\cdot \eta }_{conv}\cdot \eta_{QM}$ during the redaction of the paper.

A correction has been made to Section 3.1. Entanglement Distribution Model, Paragraph 2:

The correct equation is $\eta^{Alice}=\eta^{Bob}=\eta_{src}{\cdot \eta }_{conv}\cdot \eta_{QM}$.

**Figure Correction**

In the original publication [1], there was a mistake in Figure 5a Rate of entangled photon pairs $\sigma_{elem}^{sat}$ (*t*) (Equation (4)) received at the MéO station (Calern) and Palaiseau quantum ground stations distributed over time during the satellite pass as published. In the online paper, the authors claimed that this plot was obtained by calculating the quantity $\sigma_{elem}^{sat}(Time)={R_{src}\cdot \;\eta }_{elem}^{sat}\;(Time)\;,$ with ${\;\eta }_{elem}^{sat}\;\left(Time\right)=\eta_{src}{\cdot \eta }_{A}{\cdot \eta }_{B}{\;\cdot \eta }_{QM}^{2}\;\left(t_{k}\right)$. After publication, the authors realized that the plotted curve was actually only $\eta_{A}{\cdot \eta }_{B}$, without $\eta_{src}{\;\cdot \eta }_{QM}^{2}\;\left(t_{k}\right)$.

The corrected Figure 5a rate of entangled photon pairs $\sigma_{elem}^{sat}(t)$ (Equation (4)) received at the MéO station (Calern) and Palaiseau quantum ground stations distributed over time during the satellite pass appears below.

The authors state that the scientific conclusions are unaffected. This correction was approved by the Academic Editor. The original publication has also been updated.

## Figures and Tables

**Figure 5:**
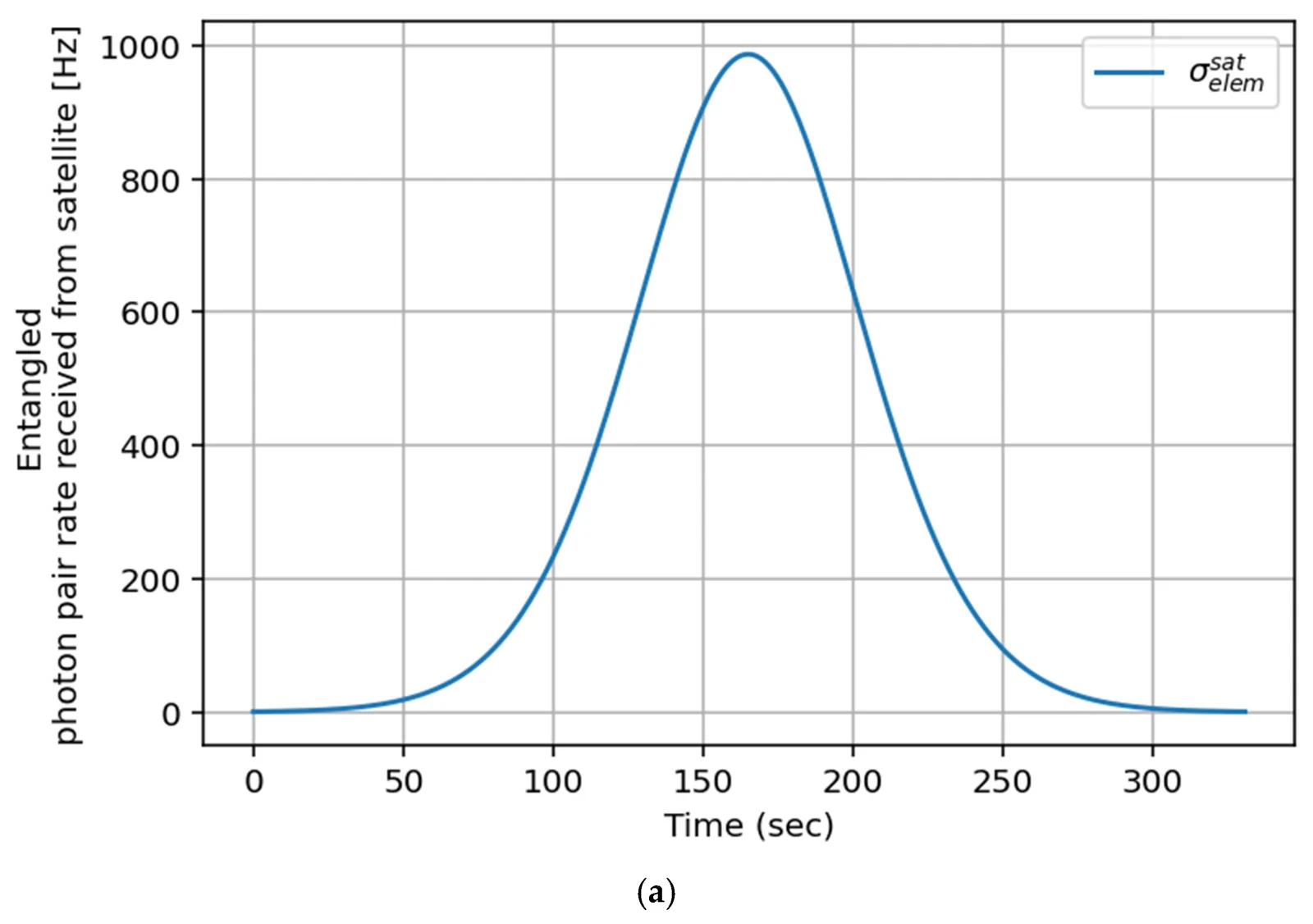
(**a**) Rate of entangled photon pairs $\sigma_{elem}^{sat}(t)$ (Equation (4)) received at the MéO station (Calern) and Palaiseau quantum ground stations distributed over time during the satellite pass.
